# Variations in risk and protective factors for life satisfaction and mental wellbeing with deprivation: a cross-sectional study

**DOI:** 10.1186/1471-2458-12-492

**Published:** 2012-07-02

**Authors:** Mark A Bellis, Helen Lowey, Karen Hughes, Lynn Deacon, Jude Stansfield, Clare Perkins

**Affiliations:** 1 Centre for Public Health, Liverpool John Moores University, 15-21 Webster Street, Liverpool, L3 2ET, UK; 2 Blackburn with Darwen Care Trust Plus, Guide Business Centre, School Lane, Blackburn, BB1 2QH, UK; 3 Independent Public Health Specialist, Manchester, UK

**Keywords:** Wellbeing, Life satisfaction, Deprivation, Smoking, Alcohol, Physical activity, Ethnicity, Health

## Abstract

**Background:**

Improving life satisfaction (LS) and mental wellbeing (MWB) is important for better public health. Like other health issues, LS and MWB are closely related to deprivation (i.e. lack of resources). Developing public health measures that reduce inequalities in wellbeing requires an understanding of how factors associated with high and low LS and MWB vary with deprivation. Here, we examine such variations and explore which public health measures are likely to improve wellbeing while reducing related inequalities.

**Methods:**

A self-administered questionnaire measuring LS and MWB was used with a cross-sectional sample of adults from the North West of England (n = 15,228). Within deprivation tertiles, analyses examined how demographics, health status, employment, relationships and behaviours (alcohol, tobacco, physical exercise) were associated with LS and MWB.

**Results:**

Deprivation was strongly related to low LS and MWB with, for instance, 17.1 % of the most deprived tertile having low LS compared to 8.9 % in the most affluent. After controlling for confounders, across all deprivation tertiles, better self-assessed health status and being in a relationship were protective against low LS and MWB. Unemployment increased risks of low LS across all tertiles but only risks of low MWB in the deprived tertile. For this tertile, South Asian ethnicity and higher levels of exercise were protective against low MWB. In the middle tertile retired individuals had a reduced risk of low MWB and an increased chance of high LS even in comparison to those in employment. Alcohol’s impact on LS was limited to the most deprived tertile where heavy drinkers were at most risk of poor outcomes.

**Conclusions:**

In this study, positive outcomes for LS and MWB were strongly associated with lower deprivation and good health status. Public health measures already developed to promote these issues are likely to improve LS and MWB. Efforts to increase engagement in exercise are also likely to have positive impacts, particularly in deprived communities. The development of future initiatives that address LS and MWB must take account of variations in their risk and protective factors at different levels of deprivation.

## Background

Mental wellbeing has been defined as: “a dynamic state in which the individual is able to develop their potential, work productively and creatively, build strong and positive relationships with others and contribute to their community. It is enhanced when an individual is able to fulfil their personal and social goals and achieve a sense of purpose in society” [1]. Individuals’ wellbeing is affected by a combination of distal and proximal factors. Thus, both good maternal health and nurturing parent–child relationships in the first few years of life have been associated with improved wellbeing across the life course [2-5]. Equally, poor parenting, adverse childhood experiences and poverty have all been linked to poorer childhood, adolescent and adult health and wellbeing [6-10]. As adults, proximal factors known to be strongly related to wellbeing include ecological measures such as deprivation, housing environments, green space access and community safety [3,11-13], as well as a variety of individual measures such as social integration and relationships, employment opportunities, attitudes and beliefs, education, and health status [14-17].

**Table 1:** Relationship between life satisfaction and demographic, economic, health and behavioural factors stratified by deprivation tertile

	**Tertile Level**	**Deprived**					**Middle**					**Affluent**					**P**^**2**^
	**Mental wellbeing**	**n**	**Low**	**Moderate**	**High**	**P**	**n**	**Low**	**Moderate**	**High**	**P**	**n**	**Low**	**Moderate**	**High**	**P**	
All		5280	21.0	60.8	18.1		5589	17.4	61.4	21.2		4342	12.8	64.6	22.6		***
Age	18-24	473	16.7	67.2	16.1	***	375	14.7	63.2	22.1	***	176	10.8	65.3	23.9	0.191	0.052
	25-39	1271	20.1	58.1	21.8		1050	13.2	62.4	24.4		630	14.0	60.3	25.7		***
	40-54	1180	24.3	58.8	16.9		1170	19.1	60.9	20.0		912	11.7	65.9	22.4		***
	55-64	804	21.9	60.9	17.2		935	19.3	57.5	23.2		814	12.7	63.3	24.1		***
	65 plus	1552	20.2	62.5	17.3		2059	18.2	62.6	19.2		1810	13.1	66.1	20.8		***
Gender	Female	3160	20.8	61.1	18.1	0.821	3335	17.3	61.8	20.9	0.741	2689	13.6	63.8	22.6	0.109	***
	Male	2120	21.4	60.3	18.3		2254	17.6	60.8	21.7		1653	11.4	66.0	22.6		***
Employment	Employed	1562	14.1	62.4	23.4	***	2099	13.6	62.5	23.9	***	1687	9.8	64.9	25.3	***	**
	Unemployed	1134	33.2	53.2	13.7		651	29.0	56.4	14.6		272	23.5	58.8	17.6		*
	FT Education	111	16.2	60.4	23.4		97	10.3	58.8	30.9		45	6.7	60.0	33.3		0.346
	Domestic/other	628	18.3	64.3	17.4		363	19.3	60.9	19.8		230	16.1	63.0	20.9		0.615
	Retired	1845	20.7	63.0	16.4		2379	17.6	62.0	20.5		2108	13.5	65.5	21.0		***
Partner status	No Partner	2466	25.5	59.9	14.5	***	2162	23.0	59.1	17.9	***	1346	18.0	62.8	19.2	***	***
	Partner employed	1244	14.4	62.6	23.0		1755	12.3	62.7	25.0		1562	9.7	65.4	24.9		**
	Partner not employed	1570	19.2	60.8	20.0		1672	15.5	62.9	21.6		1434	11.2	65.6	23.2		***
Ethnicity	White	4665	22.1	60.7	17.2	***	5353	17.4	61.6	20.9	0.106	4212	12.8	64.6	22.5	0.750	***
	South Asian	427	12.2	59.0	28.8		162	16.0	54.3	29.6		90	8.9	64.4	26.7		0.436
	Black, mixed, other	188	15.4	68.1	16.5		74	17.6	58.1	24.3		40	12.5	67.5	20.0		0.554
Health status	Very good	1175	8.7	60.8	30.6	***	1433	7.8	57.6	34.6	***	1297	5.9	64.2	29.9	***	**
	Good	1890	16.4	66.3	17.2		2170	14.6	66.0	19.4		1751	10.5	65.7	23.8		***
	Fair	1455	26.7	59.1	14.2		1423	24.0	61.3	14.7		1010	20.8	64.4	14.9		*
	Bad	599	38.6	53.1	8.3		450	34.7	54.2	11.1		231	27.3	62.8	10.0		*
	Very bad	161	49.7	40.4	9.9		113	41.6	48.7	9.7		53	39.6	54.7	5.7		0.351
Smoking status	Never smoked	2158	19.6	60.8	19.6	***	2658	17.2	60.5	22.2	***	2449	12.0	63.0	25.0	***	***
	Ex occasional	257	18.3	68.1	13.6		267	22.1	62.2	15.7		191	13.1	71.2	15.7		0.142
	Ex daily	794	17.6	64.4	18.0		1138	13.8	67.0	19.2		872	11.0	68.9	20.1		**
	Occasional	131	16.8	60.3	22.9		140	17.1	58.6	24.3		95	15.8	71.6	12.6		0.206
	Daily	1940	24.7	58.5	16.8		1386	19.8	58.4	21.7		735	16.9	62.4	20.7		***
Exercise days/week	6 to 7	941	12.0	56.7	31.2	***	1290	10.4	57.4	32.2	***	965	8.9	56.1	35.0	***	0.145
	3 to 5	1138	14.6	65.1	20.3		1388	12.2	63.0	24.9		1103	8.2	66.1	25.7		***
	1 to 2	785	20.9	63.7	15.4		965	17.8	66.5	15.6		842	13.8	68.8	17.5		**
	None	2416	27.6	59.4	12.9		1946	25.6	60.3	14.1		1432	18.3	66.9	14.8		***
Alcohol consumption category	Lower risk	1433	16.8	63.4	19.7	***	2051	13.0	63.8	23.2	***	1890	10.6	66.6	22.8	**	***
	Abstainer	2884	22.2	59.8	17.9		2435	19.7	59.2	21.1		1662	14.9	62.3	22.8		***
	Not usual drinker	395	21.0	60.3	18.7		467	22.3	62.7	15.0		301	15.9	60.8	23.3		*
	Increasing risk	417	23.7	61.4	14.9		518	18.5	60.8	20.7		418	10.5	69.1	20.3		***
	Higher risk	151	31.1	54.3	14.6		118	22.0	60.2	17.8		71	18.3	57.7	23.9		0.152

P^2^ = probability relating to comparison between tertiles within each variable category. P *** <0.001; ** < 0.01; * < 0.05. n = number; FT = Full Time.

**Table 2:** Relationship between mental wellbeing and demographic, economic, health and behavioural factors stratified by deprivation tertile

**Tertile Level**		**Deprived**					**Middle**					**Affluent**					**P**^**2**^
**Mental wellbeing**		**n**	**Low**	**Moderate**	**High**	**P**	**n**	**Low**	**Moderate**	**High**	**P**	**n**	**Low**	**Moderate**	**High**	**P**	
All		5280	21.0	60.8	18.1		5589	17.4	61.4	21.2		4342	12.8	64.6	22.6		***
Age	18-24	473	16.7	67.2	16.1	***	375	14.7	63.2	22.1	***	176	10.8	65.3	23.9	0.191	0.052
	25-39	1271	20.1	58.1	21.8		1050	13.2	62.4	24.4		630	14.0	60.3	25.7		***
	40-54	1180	24.3	58.8	16.9		1170	19.1	60.9	20.0		912	11.7	65.9	22.4		***
	55-64	804	21.9	60.9	17.2		935	19.3	57.5	23.2		814	12.7	63.3	24.1		***
	65 plus	1552	20.2	62.5	17.3		2059	18.2	62.6	19.2		1810	13.1	66.1	20.8		***
Gender	Female	3160	20.8	61.1	18.1	0.821	3335	17.3	61.8	20.9	0.741	2689	13.6	63.8	22.6	0.109	***
	Male	2120	21.4	60.3	18.3		2254	17.6	60.8	21.7		1653	11.4	66.0	22.6		***
Employment	Employed	1562	14.1	62.4	23.4	***	2099	13.6	62.5	23.9	***	1687	9.8	64.9	25.3	***	**
	Unemployed	1134	33.2	53.2	13.7		651	29.0	56.4	14.6		272	23.5	58.8	17.6		*
	FT Education	111	16.2	60.4	23.4		97	10.3	58.8	30.9		45	6.7	60.0	33.3		0.346
	Domestic/other	628	18.3	64.3	17.4		363	19.3	60.9	19.8		230	16.1	63.0	20.9		0.615
	Retired	1845	20.7	63.0	16.4		2379	17.6	62.0	20.5		2108	13.5	65.5	21.0		***
Partner status	No Partner	2466	25.5	59.9	14.5	***	2162	23.0	59.1	17.9	***	1346	18.0	62.8	19.2	***	***
	Partner employed	1244	14.4	62.6	23.0		1755	12.3	62.7	25.0		1562	9.7	65.4	24.9		**
	Partner not employed	1570	19.2	60.8	20.0		1672	15.5	62.9	21.6		1434	11.2	65.6	23.2		***
Ethnicity	White	4665	22.1	60.7	17.2	***	5353	17.4	61.6	20.9	0.106	4212	12.8	64.6	22.5	0.750	***
	South Asian	427	12.2	59.0	28.8		162	16.0	54.3	29.6		90	8.9	64.4	26.7		0.436
	Black, mixed, other	188	15.4	68.1	16.5		74	17.6	58.1	24.3		40	12.5	67.5	20.0		0.554
Health status	Very good	1175	8.7	60.8	30.6	***	1433	7.8	57.6	34.6	***	1297	5.9	64.2	29.9	***	**
	Good	1890	16.4	66.3	17.2		2170	14.6	66.0	19.4		1751	10.5	65.7	23.8		***
	Fair	1455	26.7	59.1	14.2		1423	24.0	61.3	14.7		1010	20.8	64.4	14.9		*
	Bad	599	38.6	53.1	8.3		450	34.7	54.2	11.1		231	27.3	62.8	10.0		*
	Very bad	161	49.7	40.4	9.9		113	41.6	48.7	9.7		53	39.6	54.7	5.7		0.351
Smoking status	Never smoked	2158	19.6	60.8	19.6	***	2658	17.2	60.5	22.2	***	2449	12.0	63.0	25.0	***	***
	Ex occasional	257	18.3	68.1	13.6		267	22.1	62.2	15.7		191	13.1	71.2	15.7		0.142
	Ex daily	794	17.6	64.4	18.0		1138	13.8	67.0	19.2		872	11.0	68.9	20.1		**
	Occasional	131	16.8	60.3	22.9		140	17.1	58.6	24.3		95	15.8	71.6	12.6		0.206
	Daily	1940	24.7	58.5	16.8		1386	19.8	58.4	21.7		735	16.9	62.4	20.7		***
Exercise days/week	6 to 7	941	12.0	56.7	31.2	***	1290	10.4	57.4	32.2	***	965	8.9	56.1	35.0	***	0.145
	3 to 5	1138	14.6	65.1	20.3		1388	12.2	63.0	24.9		1103	8.2	66.1	25.7		***
	1 to 2	785	20.9	63.7	15.4		965	17.8	66.5	15.6		842	13.8	68.8	17.5		**
	None	2416	27.6	59.4	12.9		1946	25.6	60.3	14.1		1432	18.3	66.9	14.8		***
Alcohol consumption category	Lower risk	1433	16.8	63.4	19.7	***	2051	13.0	63.8	23.2	***	1890	10.6	66.6	22.8	**	***
	Abstainer	2884	22.2	59.8	17.9		2435	19.7	59.2	21.1		1662	14.9	62.3	22.8		***
	Not usual drinker	395	21.0	60.3	18.7		467	22.3	62.7	15.0		301	15.9	60.8	23.3		*
	Increasing risk	417	23.7	61.4	14.9		518	18.5	60.8	20.7		418	10.5	69.1	20.3		***
	Higher risk	151	31.1	54.3	14.6		118	22.0	60.2	17.8		71	18.3	57.7	23.9		0.152

P^2^ = probability relating to comparison between tertiles within each variable category. P *** <0.001; ** < 0.01; * < 0.05. n = number; FT = Full Time.

**Table 3:** Adjusted odds for low or high life satisfaction compared with moderate levels by deprivation strata

	**Tertile Level**	**Deprived**						**Middle**						**Affluent**					
	**Life satisfaction**	**Low**			**High**			**Low**			**High**			**Low**			**High**		
		**AOR**	**95%CIs**	**P**	**AOR**	**95%CIs**	**P**	**AOR**	**95%CIs**	**P**	**AOR**	**95%CIs**	**P**	**AOR**	**95%CIs**	**P**	**AOR**	**95%CIs**	**P**
Age	18-24 (Ref)																		
	25-39	0.93	0.68-1.28	ns	0.93	0.71-1.21	ns	1.02	0.66-1.58	ns	0.83	0.62-1.11	ns	0.96	0.50-1.86	ns	0.94	0.62-1.40	ns
	40-54	0.98	0.71-13.5	ns	0.94	0.71-1.25	ns	1.30	0.85-1.99	ns	0.68	0.51-0.92	*	0.66	0.34-1.27	ns	0.95	0.64-1.41	ns
	55-64	0.59	0.40-0.86	**	1.13	0.81-1.57	ns	0.68	0.43-1.09	ns	1.06	0.77-1.44	ns	1.00	0.51-1.96	ns	1.17	0.77-1.78	ns
	65 plus	0.47	0.28-0.79	**	1.43	0.94-2.17	ns	0.68	0.38-1.20	ns	1.12	0.77-1.61	ns	0.71	0.34-1.51	ns	1.41	0.89-2.22	ns
Employment	Employed (Ref)																		
	Unemployed	1.71	1.34-2.19	***	1.10	0.88-1.37	ns	2.07	1.58-2.72	***	1.13	0.88-1.44	ns	1.62	1.09-2.42	*	1.09	0.78-1.53	ns
	FT Education	0.73	0.37-1.45	ns	0.83	0.50-1.38	ns	1.11	0.48-2.56	ns	1.49	0.91-2.43	ns	0.47	0.10-2.20	ns	0.61	0.30-1.26	ns
	Domestic/other	2.00	1.51-2.65	***	1.53	1.21-1.92	***	1.65	1.15-2.35	*	1.07	0.82-1.40	ns	1.45	0.89-2.38	ns	1.34	0.98-1.82	ns
	Retired	1.09	0.71-1.67	ns	1.26	0.90-1.77	ns	1.05	0.68-1.60	ns	1.38	1.06-1.79	*	0.78	0.49-1.22	ns	1.23	0.93-1.61	ns
Partner status & employment Ethnicity	No Partner (Ref)																		
	Partner employed	0.52	0.41-0.67	***	1.27	1.05-1.53	*	0.54	0.42-0.69	***	1.49	1.25-1.78	***	0.57	0.41-0.78	**	1.13	0.92-1.38	ns
	Partner not employed	0.79	0.65-0.95	*	1.27	1.09-1.49	**	0.72	0.59-0.89	***	1.39	1.20-1.62	***	0.72	0.55-0.95	*	1.28	1.08-1.52	**
	White (Ref)																		
	South Asian	0.97	0.68-1.38	ns	1.19	0.92-1.54	ns	1.05	0.62-1.80	ns	0.90	0.61-1.33	ns	1.37	0.64-2.93	ns	1.00	0.62-1.61	ns
	Black, mixed, other	1.40	0.94-2.08	ns	0.70	0.47-1.06	ns	1.57	0.77-3.22	ns	0.72	0.40-1.28	ns	0.31	0.04-2.32	ns	1.39	0.72-2.69	ns
Health status	Very good (Ref)																		
	Good	1.29	0.98-1.70	ns	0.48	0.40-0.57	***	1.23	0.92-1.65	ns	0.50	0.43-0.58	***	1.55	1.06-2.27	*	0.43	0.37-0.51	***
	Fair	2.43	1.83-3.24	***	0.41	0.34-0.50	***	2.25	1.66-3.06	***	0.39	0.33-0.48	***	3.32	2.24-4.93	***	0.27	0.22-0.33	***
	Bad	5.23	3.76-7.27	***	0.38	0.28-0.51	***	4.92	3.45-7.03	***	0.22	0.16-0.31	***	6.78	4.12-11.14	***	0.26	0.18-0.38	***
	Very bad	10.32	6.59-16.17	***	0.38	0.22-0.68	**	10.77	6.43-18.04	***	0.40	0.22-0.74	**	7.57	3.69-15.52	***	0.09	0.03-0.25	***
Smoking status	Never smoked (Ref)																		
	Ex occasional	0.69	0.43-1.09	ns	1.06	0.79-1.44	ns	1.14	0.74-1.75	ns	0.97	0.72-1.30	ns	0.90	0.49-1.67	ns	0.84	0.60-1.17	ns
	Ex daily	1.22	0.95-1.57	ns	1.05	0.85-1.28	ns	1.30	1.02-1.65	*	1.13	0.97-1.33	ns	1.50	1.12-2.00	**	1.14	0.96-1.36	ns
	Occasional	2.26	1.40-3.64	**	1.35	0.87-2.10	ns	1.20	0.69-2.10	ns	1.06	0.71-1.57	ns	2.30	1.22-4.31	*	0.97	0.61-1.56	ns
	Daily	1.19	0.98-1.44	ns	0.91	0.77-1.08	ns	1.38	1.11-1.72	**	0.95	0.81-1.12	ns	1.43	1.06-1.93	*	0.88	0.72-1.07	ns
Exercise days/week	6 to 7 (Ref)																		
	3 to 5	0.95	0.71-1.27	ns	0.68	0.56-0.83	***	0.71	0.53-0.95	*	0.78	0.66-0.93	**	1.10	0.76-1.58	ns	0.91	0.75-1.10	ns
	1 to 2	0.97	0.71-1.32	ns	0.46	0.36-0.58	***	0.94	0.71-1.26	ns	0.61	0.50-0.74	***	1.08	0.74-1.57	ns	0.82	0.66-1.00	*
	None	1.30	1.01-1.67	*	0.66	0.55-0.80	***	1.15	0.90-1.48	ns	0.80	0.67-0.95	*	1.13	0.80-1.58	ns	0.89	0.73-1.08	ns
Alcohol consumption category	Lower risk (Ref)																		
	Abstainer	1.30	1.05-1.60	*	1.05	0.90-1.24	ns	1.03	0.84-1.28	ns	1.01	0.87-1.16	ns	0.94	0.72-1.22	ns	1.06	0.91-1.24	ns
	Not usual drinker	1.51	1.09-2.08	*	0.94	0.71-1.25	ns	0.99	0.70-1.41	ns	1.15	0.92-1.45	ns	1.04	0.67-1.61	ns	1.00	0.76-1.31	ns
	Increasing risk	1.56	1.14-2.14	**	0.81	0.61-1.07	ns	1.27	0.92-1.74	ns	0.90	0.72-1.13	ns	0.98	0.65-1.48	ns	1.00	0.79-1.27	ns
	Higher risk	1.93	1.25-2.98	**	0.88	0.55-1.43	ns	1.22	0.69-2.16	ns	0.92	0.59-1.45	ns	1.64	0.76-3.53	ns	1.25	0.73-2.15	ns

P *** <0.001; ** < 0.01; * < 0.05; ns = not significant; AOR = Adjusted Odds Ratio. 95%CI = 95 % Confidence Intervals.

Ref = reference group. FT = Full Time.

**Table 4:** Adjusted odds for low or high mental wellbeing compared with moderate levels by deprivation strata

	**Tertile Levels**	**Deprived**						**Middle**						**Affluent**					
	**Mental wellbeing**	**Low**			**High**			**Low**			**High**			**Low**			**High**		
		**AOR**	**95%CIs**	**P**	**AOR**	**95%CIs**	**P**	**AOR**	**95%CIs**	**P**	**AOR**	**95%CIs**	**P**	**AOR**	**95%CIs**	**P**	**AOR**	**95%CIs**	**P**
Age	18-24 (Ref)																		
	25-39	1.42	1.05-1.93	*	1.63	1.20-2.22	**	0.92	0.63-1.34	ns	1.24	0.89-1.72	ns	1.53	0.85-2.77	ns	1.34	0.85-2.11	ns
	40-54	1.16	0.85-1.58	ns	1.50	1.09-2.08	*	1.18	0.82-1.70	ns	1.17	0.84-1.63	ns	1.01	0.56-1.82	ns	1.09	0.70-1.70	ns
	55-64	0.80	0.56-1.15	ns	2.05	1.42-2.96	***	1.25	0.85-1.84	ns	1.52	1.06-2.18	*	0.89	0.48-1.64	ns	1.33	0.84-2.13	ns
	65 plus	0.67	0.42-1.06	ns	3.15	1.94-5.11	***	1.17	0.74-1.87	ns	1.21	0.80-1.84	ns	0.58	0.30-1.14	ns	1.32	0.79-2.19	ns
Employment	Employed (Ref)																		
	Unemployed	1.51	1.21-1.89	***	0.89	0.71-1.13	ns	1.27	0.99-1.63	ns	0.87	0.67-1.15	ns	1.24	0.86-1.80	ns	1.03	0.72-1.49	ns
	FT Education	1.32	0.74-2.36	ns	1.27	0.76-2.12	ns	0.80	0.38-1.68	ns	1.53	0.90-2.59	ns	0.74	0.20-2.73	ns	1.49	0.72-3.07	ns
	Domestic/other	1.02	0.78-1.34	ns	0.74	0.57-0.96	*	1.28	0.93-1.74	ns	0.84	0.63-1.13	ns	1.33	0.88-2.00	ns	0.82	0.57-1.17	ns
	Retired	1.17	0.80-1.70	ns	0.59	0.40-0.86	**	0.68	0.49-0.95	*	1.27	0.95-1.69	ns	1.20	0.80-1.79	ns	0.93	0.69-1.26	ns
Partner status & employment Ethnicity	No Partner (Ref)																		
	Partner employed	0.68	0.55-0.85	***	1.26	1.02-1.55	*	0.57	0.46-0.70	***	1.20	0.99-1.45	ns	0.54	0.41-0.72	***	1.07	0.85-1.34	ns
	Partner not employed	0.83	0.70-0.98	*	1.22	1.02-1.46	*	0.69	0.58-0.82	***	1.06	0.89-1.26	ns	0.69	0.55-0.86	**	1.12	0.92-1.36	ns
	White (Ref)																		
	South Asian	0.61	0.43-0.86	**	1.74	1.33-2.29	***	1.02	0.64-1.64	ns	1.75	1.19-2.57	**	0.63	0.29-1.36	ns	1.00	0.60-1.67	ns
	Black, mixed, other	0.59	0.38-0.91	*	0.88	0.57-1.34	ns	1.34	0.69-2.60	ns	1.12	0.62-2.02	ns	0.84	0.32-2.24	ns	0.83	0.37-1.87	ns
Health status	Very good (Ref)																		
	Good	1.69	1.32-2.16	***	0.54	0.45-0.65	***	1.46	1.15-1.86	**	0.50	0.43-0.59	***	1.78	1.33-2.38	***	0.84	0.70-1.00	*
	Fair	2.90	2.23-3.76	***	0.54	0.43-0.67	***	2.32	1.80-2.99	***	0.43	0.35-0.53	***	3.40	2.50-4.64	***	0.57	0.45-0.72	***
	Bad	4.47	3.30-6.04	***	0.39	0.27-0.55	***	3.31	2.42-4.54	***	0.40	0.28-0.56	***	4.22	2.77-6.44	***	0.45	0.28-0.73	**
	Very bad	7.09	4.65-10.81	***	0.60	0.34-1.08	ns	4.35	2.71-6.96	***	0.39	0.20-0.77	**	6.92	3.63-13.16	***	0.29	0.09-0.96	*
Smoking status	Never smoked (Ref)																		
	Ex occasional	0.80	0.56-1.14	ns	0.63	0.43-0.94	*	1.27	0.91-1.77	ns	0.68	0.47-0.98	*	1.00	0.63-1.59	ns	0.58	0.38-0.88	*
	Ex daily	0.73	0.58-0.92	**	0.97	0.77-1.22	ns	0.68	0.55-0.84	***	0.83	0.69-1.00	*	0.80	0.62-1.04	ns	0.78	0.63-0.95	*
	Occasional	0.66	0.39-1.09	ns	1.28	0.82-2.01	ns	0.96	0.59-1.57	ns	1.18	0.77-1.80	ns	1.16	0.64-2.10	ns	0.44	0.23-0.82	*
	Daily	0.93	0.79-1.11	ns	1.05	0.88-1.27	ns	0.98	0.81-1.18	ns	1.17	0.99-1.40	ns	1.14	0.89-1.47	ns	0.89	0.72-1.11	ns
Exercise days/week	6 to 7 (Ref)																		
	3 to 5	1.20	0.91-1.57	ns	0.51	0.41-0.63	***	1.13	0.88-1.45	ns	0.69	0.58-0.82	***	0.80	0.58-1.10	ns	0.62	0.51-0.75	***
	1 to 2	1.67	1.26-2.20	***	0.40	0.31-0.52	***	1.46	1.13-1.88	**	0.42	0.34-0.52	***	1.18	0.86-1.60	ns	0.41	0.33-0.52	***
	None	1.67	1.31-2.12	***	0.42	0.34-0.51	***	1.80	1.44-2.26	***	0.48	0.40-0.59	***	1.20	0.90-1.60	ns	0.40	0.33-0.50	***
Alcoholconsumption category	Lower risk (Ref)																		
	Abstainer	1.19	0.99-1.42	ns	1.02	0.85-1.22	ns	1.22	1.02-1.46	*	1.13	0.96-1.32	ns	1.18	0.95-1.48	ns	1.22	1.03-1.46	*
	Not usual drinker	1.15	0.85-1.55	ns	1.09	0.81-1.48	ns	1.63	1.25-2.13	***	0.69	0.51-0.91	*	1.43	0.99-2.05	ns	1.14	0.84-1.55	ns
	Increasing risk	1.31	0.98-1.74	ns	0.77	0.56-1.06	ns	1.52	1.16-2.01	**	0.88	0.69-1.14	ns	0.97	0.68-1.39	ns	0.87	0.67-1.15	ns
	Higher risk	1.49	0.99-2.26	ns	0.94	0.57-1.56	ns	1.53	0.93-2.52	ns	0.79	0.47-1.32	ns	1.62	0.82-3.17	ns	1.19	0.66-2.15	ns

P *** <0.001; ** < 0.01; * < 0.05; ns = not significant; AOR = Adjusted Odds Ratio. 95%CI = 95 % Confidence Intervals.

Ref = reference group. FT = Full Time.

National and international policy makers are seeking effective and economic ways of improving population wellbeing [18,19]. Work on social determinants has identified the negative impact of inequitable societies on wellbeing [20-22]; although debate continues on the most appropriate definitions and mechanisms for its measurement [23,24]. The UK government has implemented a number of policy measures to make wellbeing, along with reductions in health inequalities, pan-governmental objectives [3,25,26] including introducing subjective wellbeing questions into national household surveys [27]. While providing a national measure of self-reported life satisfaction and wellbeing, to date this work has not examined how risk and protective factors for poor wellbeing vary with deprivation. Moreover, large studies on such issues elsewhere are scarce. Relatively little is also known about how policies aimed at reducing health inequalities may differentially impact on wellbeing in richer and poorer communities. However, such data are critical to ensure that existing public health services (e.g. alcohol, tobacco control, exercise initiatives) and policy specifically developed to improve wellbeing can, and does, reduce inequalities.

Here, we employ a simple but widely used scale of life satisfaction (LS) as a proxy for aspects of wellbeing such as happiness and pleasure [28] and, for mental wellbeing (MWB), an established shortened version of the Warwick-Edinburgh Mental Wellbeing Scale (SWEMWBS) [29]. Using these two different measures, LS and MWB, we examine their relationships with demographics, employment and health status. We explore how other public health priorities (here, alcohol, tobacco and exercise) are associated with low, moderate and high wellbeing. Importantly, we measure how such relationships differ between deprived, middle and affluent communities. Finally, we discuss how understanding the risk and protective factors associated with different levels of wellbeing can be used to increase LS and MWB in general and reduce inequalities in wellbeing.

## Methods

The study was undertaken across the North West of England; a European region with a population of 6,897,905 (2009 [30]) which includes a mix of affluent, deprived, urban and rural communities [31]. Survey sample size was calculated to provide estimates of LS and MWB at a sub-regional geography corresponding with local health authority areas (n = 24). The target sample was 18,500 individuals aged 16 years and over. Ethical approval was obtained from Liverpool John Moores University.

Households were selected for inclusion using a clustered random sample approach. The national Post Office Address File (a database containing all known UK addresses and postcodes) was employed as the sampling frame and lower super output areas (LSOAs) were the primary sampling units. LSOAs are standard geographical areas in England that contain approximately 1,500 residents [32]. Each LSOA has an average measure of deprivation routinely calculated across residents based on the Index of Multiple Deprivation (IMD). The IMD is a composite measure that includes 38 indicators relating to health, economic and educational status [33]. LSOAs within each health area were labelled by national quintile of IMD and a random selection of LSOAs was made for each quintile based on their proportion within the health area. Households were randomly selected within each specific LSOA resulting in a total of 30,884 households. At the point of visit, the household resident aged ≥16 years who was next to have their birthday was selected to participate in the survey. However, for this analysis of adult LS and MWB, only those aged ≥18 years are included. For the purpose of this study IMD quintile for respondents’ LSOA of residence was used as an ecological measure of respondents’ deprivation.

The survey was piloted and data collected between 1 April and 30 June 2009. Sampling times ranged from 9.00 am to 8.00 pm and sampling was undertaken on weekdays and weekends to ensure those in daytime employment or education were included. Trained researchers attempted a maximum of four visits to each selected household; after which another household was selected at random from the same cluster. Of all households selected 60.1 % participated.

Data were collected using a structured questionnaire containing 44 questions covering relationships, health, lifestyle behaviours and key demographics such as age, gender, ethnicity and postcode of residence (a full list of data items is published elsewhere [34]). Researchers explained the purpose of the survey, its confidential and anonymous nature and introduced the hand-held computer assisted self-interviewing (CASI) devices as the mechanism for data collection [35]. Where required (e.g. with some elderly respondents) researchers provided assistance with using the devices. The final sample size was 18,560.

MWB was measured using the Short Warwick-Edinburgh Mental Wellbeing Scale (SWEMWBS). This is a shortened seven-item alternative to the full version of WEMWBS which uses a series of Likert-style items asking people to describe their experience of feelings and thoughts over the last two weeks: 1) I’ve been feeling optimistic about the future; 2) I’ve been feeling useful; 3) I’ve been feeling relaxed; 4) I’ve been dealing with problems well; 5) I’ve been thinking clearly; 6) I’ve been feeling close to other people; 7) I’ve been able to make my own mind up about people [29]. Responses are scored (none of the time = 1; rarely = 2; some of the time = 3; often = 4; all the time = 5) and summed to provide a total score for each respondent. The SEMWBS has been validated against the full WEMWBS [29] and been used to measure wellbeing in UK national household surveys [36].

LS was measured using the single item question: “All things considered, how satisfied are you with your life as a whole nowadays on a scale of 1 to 10 where one is extremely dissatisfied and 10 is extremely satisfied” [37]. This is a well-established measure that is widely used in global (e.g. World Values Survey [38]), European (e.g. European Social Survey [37]); and UK (e.g. British Social Attitudes Survey [28]) population surveys. Across surveys that use this question, average LS values for UK populations have been relatively consistent. Some variations in the wording and scale of single item LS questions are used in other social surveys, yet results produced are generally similar [28].

For the purposes of analyses respondents were categorised for MWB into moderate (mean +/− one standard deviation (SD) range 23–32), low (below one SD from the mean) and high (above one SD from the mean). LS was categorised into low (scored 1–5), moderate (6–8) and high (9–10).

Employment was categorised as employed (including full-time, part-time and self- employed), unemployed (including seeking work, not seeking work for reasons of disability), retired, and domestic/other (of which those engaged in domestic duties accounted for 77.4 %). Individuals were also categorised according to whether they had a partner (here, in a long term meaningful relationship) and, if so, if that person was employed. Ethnicity was recorded using standard UK ethnic group categories [39]; however due to small numbers of some ethnic minorities it was only possible to categorise ethnicity into three groups: White, South Asian and Other (of Other, 44.4 % were Black). For health status we used a standard self-assessed five point scale from very good to very bad health (“How is your health in general? Would you say it is…”) [40]. Smoking status categorised people into five categories; never smokers, ex-occasional smokers, ex-daily smokers, current occasional smokers and current daily smokers. Exercise was measured by asking participants “In the past week, on how many days have you accumulated at least 30 minutes of moderate intensity physical activity such as brisk walking, cycling, sport, exercise, and active recreation? (do not include physical activity that may be part of your job or usual role activities)” [41].

For alcohol consumption, participants were asked “In general, how often do you drink alcoholic drinks” (response categories: never, monthly or less, once or twice a week, three or four days a week, daily or almost daily, don’t know/refused) followed by a question asking drinkers how many of a range of drink types they had consumed in the last week. Individuals who reported never drinking were categorised as abstainers and those that reported drinking monthly or less were categorised as not usual drinkers. All other drinkers were categorised based on the amount of alcohol they reported to have consumed in the past week according to UK Department of Health categories. Thus, lower risk drinkers were, for females, those that drank ≤14 units (standards UK drinks; 1 unit = 8 grams of pure alcohol) per week and, for males, ≤21 units per week. Increasing risk drinkers were those that consumed 15 to 35 units (females) and 22 to 50 units (males) per week and higher risk drinkers were those that consumed >35 (female) and >50 units (males) per week [42]. To ensure that drinking reported over the past week was representative of typical drinking behaviour, individuals were asked if last week’s drinking was higher, lower or about typical for their alcohol consumption. Only those who stated that their past weeks’ alcohol consumption was typical were included in the analysis. Those individuals not providing an age (n = 286), not answering both LS and MWB questions (n = 383) and not providing answers to all other general health, smoking or exercise questions (n = 294) were excluded from analyses.

The final sample contained 15,228 individuals who had provided answers across all variables included in analyses (82 % of all those completing the survey). Statistics utilised chi-squared for bivariate exploration of LS and MWB across the entire dataset. Sample size did not permit robust analyses within each deprivation quintile. Therefore, to examine associations with LS and MWB at different levels of deprivation the dataset was split into tertiles (affluent, n = 4346; middle, n = 5598; deprived, n = 5284). Sample sizes vary slightly between tertiles as the catchment of each was matched to national IMD ranges for quintiles 1&2, 3&4 and 5 respectively. For each tertile, multinomial logistic regression (MLR [43]) was used to identify independent relationships between LS / MWB and demographic, employment and lifestyle factors. Moderate LS / MWB was used as the reference category in order to quantify odds of low or high LS / MWB. All analyses were undertaken in PASW (Predictive Analytics Software) v18.0.

## Results

### Deprivation

Life satisfaction was strongly related to deprivation, with high LS increasing with affluence and low LS with deprivation (Table 1). This pattern was replicated across all age categories, both genders, and all categories of partner status, health status, exercise frequency and alcohol consumption. Associations between LS and deprivation were not significant for those in full time education, those of South Asian ethnicity, and current- or ex-occasional smokers. High LS peaked in affluent individuals with self-reported very good health (50.8 %), while low LS peaked in those with very bad health from the deprived tertile (54.0 %) (Table 1).

MWB also showed strong relationships with deprivation, again with high MWB increasing with affluence and low MWB increasing with deprivation (Table 2). This trend was seen in both genders and in all but the youngest age group. Exceptions to the trend included those: in full time education or domestic/other categories; of South Asian or Other ethnicity; with very bad health; who exercised most frequently; who were current- or ex-occasional smokers; and higher risk drinkers. High MWB peaked in affluent individuals who exercised 6–7 days/week (35.0 %; Table 2). As with LS, low MWB was most common among deprived participants with self-reported very bad health (49.7 %).

### Age

LS showed significant variation by age in all deprivation tertiles (Table 1). Proportions with high LS ranged from 37.6 % of those aged ≥65 years in the affluent tertile to only 19.7 % of those aged 40–54 years in the deprived tertile. While 22.6 % of 40–54 year olds had low LS in the deprived tertile, this fell to 7.8 % in affluent individuals of the same age (Table 1). MLR analyses identified older age (age ≥55) as being protective against low LS in individuals from the deprived tertile (compared to individuals with moderate LS). In the middle tertile, high LS was significantly less likely in the 40–54 year age group (Table 3). For MWB, even in bivariate analyses only deprived and middle tertiles showed significant relationships with age (Table 2). Using MLR, in deprived areas having high MWB was strongly associated with increasing age and in the middle tertile MWB peaked in those aged 55–64 years (Table 4).

### Gender

In bivariate analyses there were no relationships between gender and either LS or MWB in any deprivation tertile (Tables 1 & 2). Consequently, gender was not included in MLR analyses.

### Employment

Both LS and MWB showed significant bivariate relationships with employment status in all deprivation tertiles. Unemployed individuals were least likely to have high LS and MWB and most likely to have low LS and MWB across all strata (Tables 1 & 2). After correcting for confounding, unemployment was still associated with low LS across all tertiles (Table 3) but for MWB, significant effects were limited to the most deprived groups (Table 4). In the middle tertile retired individuals had higher LS and MWB even compared with those in employment (Tables 3 & 4).

### Partner status

Across all deprivation strata participants that had a partner, regardless of their partner’s employment status, were more likely to have high LS and MWB and less likely to have low LS and MWB than those without a partner (Tables 1 & 2). After controlling for confounding factors, these relationships remained for LS (Table 3). For MWB, while having a partner remained protective against low MWB across all tertiles, the relationship between having a partner and high MWB was only significant in the deprived tertile (Table 4). Across both LS and MWB the employment status of partners had no major impact (Tables 3 & 4).

### Ethnicity

Bivariate relationships between ethnicity and both LS and MWB were only significant in the most deprived tertile (Tables 1 & 2). After adjusting for confounding factors, ethnicity had no significant relationships with LS (Table 3). However, in the deprived tertile, South Asian and Other ethnicity were protective against low MWB (compared with White ethnicity). South Asian ethnicity also predicted high MWB in both the most deprived and middle deprivation strata (Table 4).

### Health

Both LS and MWB were strongly related to self-assessed health with better health increasing high LS and MWB and poorer health increasing low LS and MWB (Tables 1 & 2). Overall, these relationships remained in MLR, with some of the largest effects on LS and MWB status relating to health (Tables 3 & 4). Thus, the odds of having low LS were more than 10 times greater in those with very bad health than in those with very good health in both deprived and middle deprivation tertiles, and over seven times greater in the most affluent tertile (Table 3).

### Smoking

In bivariate analyses, both LS and MWB varied significantly by current and historical smoking behaviours across all deprivation strata (Tables 1 & 2). Using MLR, smoking was most strongly associated with low LS, with daily and ex-daily smokers having greater risks of low LS in both affluent and middle tertiles. Occasional smoking in deprived and affluent individuals was also associated with low LS (Table 3). High MWB was strongly associated with never smoking in affluent individuals. Ex-occasional smokers were also less likely than non-smokers to have high MWB in both middle and deprived tertiles. However, compared to non-smokers, being an ex-daily smoker was protective against low MWB in the same tertiles (Table 4).

### Exercise

In bivariate analyses, more frequent exercise was significantly associated with higher LS and MWB (Tables 1 & 2) across all deprivation tertiles. In MLR, in both middle and deprived tertiles those exercising 6–7 days/week had greatest odds of high LS. Taking no exercise was associated low LS but only in the deprived tertile (Table 3). MLR also identified a strong relationship between decreasing frequency of exercise and greater low MWB in deprived and middle tertiles. Frequent exercise was associated with increased high MWB across all tertiles (Table 4).

### Alcohol

In bivariate analyses individuals in the lower risk drinking category were least likely to have low LS across all deprivation strata (Table 1). This same pattern was seen for MWB, although a similar proportion of increasing risk drinkers reported low MWB in the most affluent tertile (Table 2). However after controlling for confounding factors (Table 3), lower risk drinking was only protective against low LS in the most deprived tertile, with no other significant relationships seen between LS and drinking behaviours. For MWB lower risk drinking had a similar protective effect but only in the middle tertile. In affluent individuals, compared with lower risk drinkers MWB only differed in abstainers, with abstainers being more likely to have high MWB (Table 4).

## Discussion

Our study is one of the largest examining LS and MWB in the UK to date. Consistent with research elsewhere [15,44], data show strong relationships between a composite measure of deprivation and low LS and MWB as well as reduced levels of high LS and MWB (Tables 1 & 2). However, while studies have largely examined risk and protective factors for low LS and MWB across the entire deprivation spectrum, here we have looked at such factors within different derivation strata and treated the transition from moderate LS and MWB to low or high levels as separate outcomes.

Our results identify different factors impacting on LS and MWB in different deprivation strata and different risk and protective factors for low or high LS and MWB (cv moderate levels). For example, a recent UK survey [27] found a U shape relationship between age and lower LS scores with the nadir for LS at 45–49 years. Here, in equivalent bivariate analyses we identified peaks in low LS at age 40–54 years in both deprived and middle tertiles but at age 55–64 years in the most affluent. Moreover after correcting for other factors, the impact of age on odds of low LS was only significant in the most deprived tertile where low LS decreased with increasing age (Table 3). Equally, in the same tertile high MWB increased significantly with age (Table 4). In deprived communities, a concentration of low LS in younger age groups may relate to individuals having more adverse childhood experiences, less access to material resources and lower aspirations for achieving life goals such as meaningful careers and personal wealth [45]. This should be considered a contributory factor in their increased risk of anti-social behaviour and violence [46]. Such effects have been identified as underlying drivers of recent disturbances in the UK involving primarily youths from the most deprived communities [47].

As with other studies we found poorer LS in those who are unemployed [27], with our results confirming unemployment (cv employment) as a predictor of low LS across all deprivation strata (Table 3). In contrast, for MWB this was only the case in the most deprived tertile (Table 4). LS is more closely related to general happiness and pleasure; both of which may rapidly reduce with unemployment at any level of affluence [48]. MWB (through SWEMWBS) includes specific elements such as dealing with problems, thinking clearly, being close to others and, making up one’s own mind about things. These factors may be more resilient to changes in employment status, especially if unemployment benefit/compensation is available and where there is family support [49]. In fact, being in a meaningful relationship with a partner was strongly related to better LS and MWB outcomes across all deprivation strata. While this is consistent with other studies [27] our results also identified that the employment status of the partner had no discernible effects on outcomes (Tables 3 & 4).

Better LS and MWB are related to improved health status as both a cause and a consequence [50]. Associations between health and LS here were such that those with very bad health (in deprived and middle tertiles) had odds of low LS more than ten times higher than individuals in very good health (10.32 and 10.77 respectively; Table 3). Equivalent odds for MWB were 7.09 and 4.35 for deprived and middle tertiles respectively. High LS and MWB (cv moderate) were also strongly related to health in all tertiles (Tables 3 & 4). However, the impact of health on LS is strongly moderated by levels of deprivation. Thus, among those with self-assessed very bad health, movement from the most deprived to most affluent tertile represents a reduction in low LS membership of 21.9 % (from 54.0 % to 32.1 %). Our results suggest that LS and MWB will benefit from health-increasing measures and vice versa. However, in order to reduce inequalities such actions need to focus disproportionately on deprived communities. The strong relationship between health and both LS and MWB may partly explain the strength of public opinion on changes to health service provision. More work is required to better understand how perceived security of access to health treatment impacts on LS and MWB [51].

As well as health status, increasing deprivation was associated with lower LS and MWB within most independent variables studied here. However, no such relationships were seen in South Asian respondents (Tables 1 & 2). Whether South Asian communities benefit through stronger community ties, family relationships, cultural or individual protective factors against low LS and MWB remains unclear [52-54]. A better understanding of the protective factors present in South Asian communities should help identify mechanisms for improving wellbeing in the broader population as well as specifically in deprived communities [55].

For tobacco, public health policy has frequently been challenged on the basis that changes in legislation to improve health would reduce access to pleasure. In 2004 the Secretary of State for Health (UK) resisted changes to smoking legislation as ‘people from these lower socioeconomic categories have very few pleasures in life and one of them they regard as smoking’ [56]. In this study, there was no suggestion that smoking was linked to improved LS (cv never smoked; Table 3). Interestingly low MWB was less likely in ex-daily smokers (deprived and middle tertiles) than even those that had never smoked (Table 4). However, the causality in relationships between smoking and MWB are still unclear and individuals with good MWB are likely to have sufficient determination to quit. Equally however, our results are consistent with no fall in MWB as a result of quitting. Elsewhere, individuals who successfully quit smoking have been found to have significantly improved well-being compared with individuals who continue to smoke [57].

Drinking within government weekly guidelines for alcohol consumption was associated with lower risks of low LS, but only in the most deprived tertile (Table 3). Here, although there were minor differences from abstainers, increasing and higher risk drinkers showed the highest risks of low LS (Table 3), supporting previous studies identifying strong relationships between alcohol use and LS [58,59]. Alcohol consumption is frequently used as a form of self-medication for stress with such users often increasing consumption levels as they develop greater tolerance [60]. Consequently, higher risk drinking may be a result as well as a cause of poorer LS [58]. Importantly, the risks of alcohol-related diseases associated with any specific level of consumption appear to be higher in poorer communities [61]. Our results suggest that the impact of alcohol on LS may also be stronger in poorest communities. Lower risk drinking was protective against low MWB in the middle tertile (Table 4); even compared to not usual drinkers and abstainers [62]. As community engagement is related to MWB, more work is needed to identify whether non-drinkers can successfully engage in UK communities where socialising can be based around alcohol consumption [63].

Exercise had one of the most wide-ranging impacts on both LS and MWB (Tables 3 & 4). Importantly, higher levels of exercise were protective against low MWB in the deprived tertile but not in the affluent. The UK Chief Medical Officers released advice on exercise levels for different age groups and a series of initiatives have been established to encourage uptake (e.g. change4life, Physical Exercise Responsibility Deal) [64]. Other policy changes have also placed public health leadership within local authorities with responsibility for cycling, public- and school-based sports facilities, and parks and green space access and security [25]. With a strong evidence base to support causal relationships [65], this study suggests that physical exercise may be particularly beneficial for wellbeing in deprived communities and may be an important element in addressing wellbeing inequalities.

Although this is one of the largest studies of LS and MWB in the UK to date, it has a number of limitations. Using residential address as the sampling unit meant that an individual compliance rate and therefore sample representativeness could not be calculated. However, CASI and stratified residence sampling were used to improve compliance with approximately two thirds of all residences identified in the initial sample participating in the survey. Moreover, while we have no nationally comparable measures for MWB, a recent national survey using a version of the LS scale employed here [27] reported an average LS score of 7.4 (cv 7.57 here). SWEMWBS has not been extensively used in the general population and requires further validation to better understand the precise nature of the metric and its relationship with population mental health. For deprivation, individuals were assigned to quintiles based on their LSOA of residence. While this ecological technique is widely used it can mask individual-level differences within each LSOA. Moreover, despite a large sample size there were insufficient individuals from ethnic minorities to examine the LS and MWB characteristics of smaller ethnic groups. Using a cross-sectional survey we were not able to identify causal relationships but only associations between wellbeing, life satisfaction and deprivation, health and lifestyle factors. This is a limitation in the application of the findings to the development and implementation of measures to improve wellbeing. However, our cross-sectional data still identifies the characteristics of communities and individuals most likely to suffer from poor wellbeing; and thus where interventions and longitudinal studies may best be targeted. Finally, although we have examined how relationships between LS/MWB and behaviour (e.g. smoking and alcohol use) differ with deprivation our study was not able to identify if motivations for such behaviours also vary. Thus, in some communities, more than others, substance use may function as a coping strategy. Behaviours such as alcohol consumption and smoking may themselves be the result of early life or environmental stressors but examining such issues was not possible in this study [66].

## Conclusions

With an ageing demographic, an increasing burden of ill health and fewer resources available to meet the health needs of populations in general, governments must address wellbeing alongside established measures of population health and economic success. However, in common with these established measures it is critical that efforts to improve wellbeing also reduce associated inequalities. Our results have identified a strong relationship between deprivation and both LS and MWB. In deprived areas at least, low LS appears to decease in older age groups along with high MWB increasing. While deprived older populations are often seen as some of the most vulnerable, improving wellbeing in deprived communities may require a disproportionate focus on those under 54 years and especially those in later adolescence. A continued failure to tackle poor LS, especially in deprived adolescents, contributes to increased risks of anti-social behaviour. While ethnic minority populations often suffer poorer physical health outcomes [3], factors associated with South Asian ethnicity may offer protection against some of the negative impacts of poverty on MWB. Such factors require further study to identify any protective elements that are transferable to other communities.

Results from this study are also pertinent to the range of established public health services designed to tackle tobacco, alcohol and obesity. Thus, we found no evidence to support smoking increasing LS or MWB, with daily smokers typically having greater levels of low LS (Table 3). While we identified some negative impacts of increasing and higher risk drinking on LS and MWB, more work needs to be undertaken to understand the LS and MWB related needs of abstainers and infrequent drinkers who can be marginalised as a result of their consumption choices. Public health initiatives to increase physical exercise may be best placed to have an unequivocally positive impact on LS and MWB across all, and especially deprived, communities. Such initiatives are increasingly considered as measures not only for those with physical health needs (e.g. weight loss) but also those with poor LS and MWB. However while such services may help, the profoundly negative impacts of deprivation mean that major improvements in LS and MWB require a disproportionate focus on the poorest communities and continued efforts to address broader health and social inequalities.

## Competing interests

The author(s) declare that they have no competing interests.

## Authors’ contributions

MAB directed the study, analysed the data and wrote the manuscript. HL and KH analysed data and co-wrote the manuscript. LD contributed to the data collection, study methodology and edited the manuscript. JS and CP contributed to literature review and manuscript editing. All authors read and approved the final manuscript.

## Author’s information

JS independent consultant on public health and mental wellbeing.

## Pre-publication history

The pre-publication history for this paper can be accessed here:

http://www.biomedcentral.com/1471-2458/12/492/prepub
